# The Efficacy of Acupuncture on Tic Disorders in Children: A Retrospective and Propensity Score-Matched Study

**DOI:** 10.3389/fped.2021.745212

**Published:** 2021-11-04

**Authors:** Hai-Zhen You, Yi-Fang Zhou, Ping-Bo Yu, Jing Xie, Jia Chen, Ji-Jun Li, Guang-Hai Wang, Ke-Xing Sun

**Affiliations:** ^1^Department of Traditional Chinese Medicine, Shanghai Children's Medical Center, Shanghai Jiao Tong University School of Medicine, Shanghai, China; ^2^Department of Traditional Chinese Medicine, Shanghai University of Traditional Chinese Medicine, Shanghai, China; ^3^Department of Developmental and Behavioral Pediatrics, Pediatric Translational Medicine Institute, Shanghai Children's Medical Center, Shanghai Jiao Tong University of Medicine, Shanghai, China

**Keywords:** conventional treatment, tic disorders, vocal disorders, acupuncture—therapy, propensity analyses

## Abstract

**Background :** Acupuncture has been considered as a complementary or alternative therapy for children with tic disorders (TD), but its efficacy remains largely unknown. This study retrospectively examined the efficacy of acupuncture treatment for TD in children over the course of 12 weeks.

**Methods:** Data were collected from Traditional Chinese Medicine clinics in a public pediatric hospital in Shanghai between June 2020 and March 2021. A total of 250 patients with TD were included in the study, with 122 patients exposed to acupuncture therapy combined with conventional treatment (observation group), and 128 patients exposed to conventional treatment alone (control group). Propensity score matching analyses were used to balance baseline characteristics, resulting in 78 matched patients for each group. Reductions in the Yale Global Tic Severity Scale (YGTSS) total score were analyzed in the two groups after 12 weeks of treatment.

**Results:** The two groups reached equilibrium in terms of baseline demographic characteristics and YGTSS total score after the propensity score matching (*P* > 0.05). Compared to the control group, the reduction in the YGTSS total score after 12 weeks of treatment was greater for the observation group (OR = 2.94, 95% CI: 1.03, 8.39, *P* = 0.04), and this association was stronger for patients who had significant vocal tics (β = 0.29, 95% CI: 0.88, 2.68, *P* = 0.001). The clinical efficacy for the observation group was significantly better than the control group.

**Conclusions:** We provided preliminary evidence supporting the therapeutic effect of acupuncture for TD in children. Hence, our findings indicate that acupuncture could be an adjuvant treatment efficacious for TD in children, especially for vocal tics.

## Introduction

Tic disorders (TD) are neuropsychiatric disorders characterized by the presence of involuntary contractions of muscle groups that result in motor movements or verbal utterances and sounds. The prevalence of TD in China is ~6.1%, and the ratio of males to females is ~2–3:1 (1). TD and accompanied comorbidities can lead to decreased quality of life and dysfunction, including emotional instability and poor sleep quality (2). Although comprehensive behavioral intervention for tics (CBIT) is recommended as the first-line treatment for children with TD, its accessibility is limited due to higher demands on expertise and lacking of experience and confidence among clinicians and parents (3, 4). Moreover, scarce evidence endorses the use of deep brain stimulation (DBS) in the treatment of tic disorders (5). Additionally, pharmaceutical treatments such as Aripiprazole and other dopamine receptor blockers often induce side effects, including sedation, somnolence, increased appetite, and thus often have limited acceptance and compliance (6). Consequently, it is critical to develop complementary or alternative therapy, and determine the efficacy in favor of better and more comprehensive treatment for children with TD.

Acupuncture has been implemented in China for more than 2,000 years. This treatment modality has many advantages as compared with drug therapy in the treatment of certain diseases, in terms of safety, effectiveness, convenience, and fewer side effects. Therefore, acupuncture is considered as a valid and effective complementary or alternative therapy (7), and commonly used to prevent and treat neurological and psychological illnesses (8). However, empirical evidence are required to evaluate the efficacy of this treatment modality for TD (9). To our best knowledge, sporadic studies revealed beneficial effects of acupuncture in the treatment of TD in children, even more efficacious than pharmaceutical medications alone, whereas these findings should be confirmed through more investigations due to small sample sizes and poor design quality (10).

Traditional Chinese acupuncture theory states that health is achieved by maintaining an uninterrupted flow of Qi. The needling sensation of Deqi during acupuncture is the key factor influencing acupuncture outcome. Emerging neuroimaging studies provided new evidence for the neuromodulation effect of Deqi during acupuncture in patients with ischemic stroke (11) and depression (12), which might be extended to children with TD.

Propensity analysis has been widely applied in epidemiology with the development of hospital informatization. This statistical approach can effectively control for confounding bias, and make groups comparable, especially suitable for examining the treatment efficacy in real clinical settings. In the current study, a propensity score matching (PSM) was performed to balance patients' baseline data, and the clinical efficacy of acupuncture treatment combined with conventional treatment compared to conventional treatment alone was observed in patients with TD after adjusting for confounding factors, referring to reporting and guidelines in propensity score analysis (13). Our study would provide additional evidence on the efficacy of acupuncture for TD in children.

## Methods

### Participants

**Figure 1:**
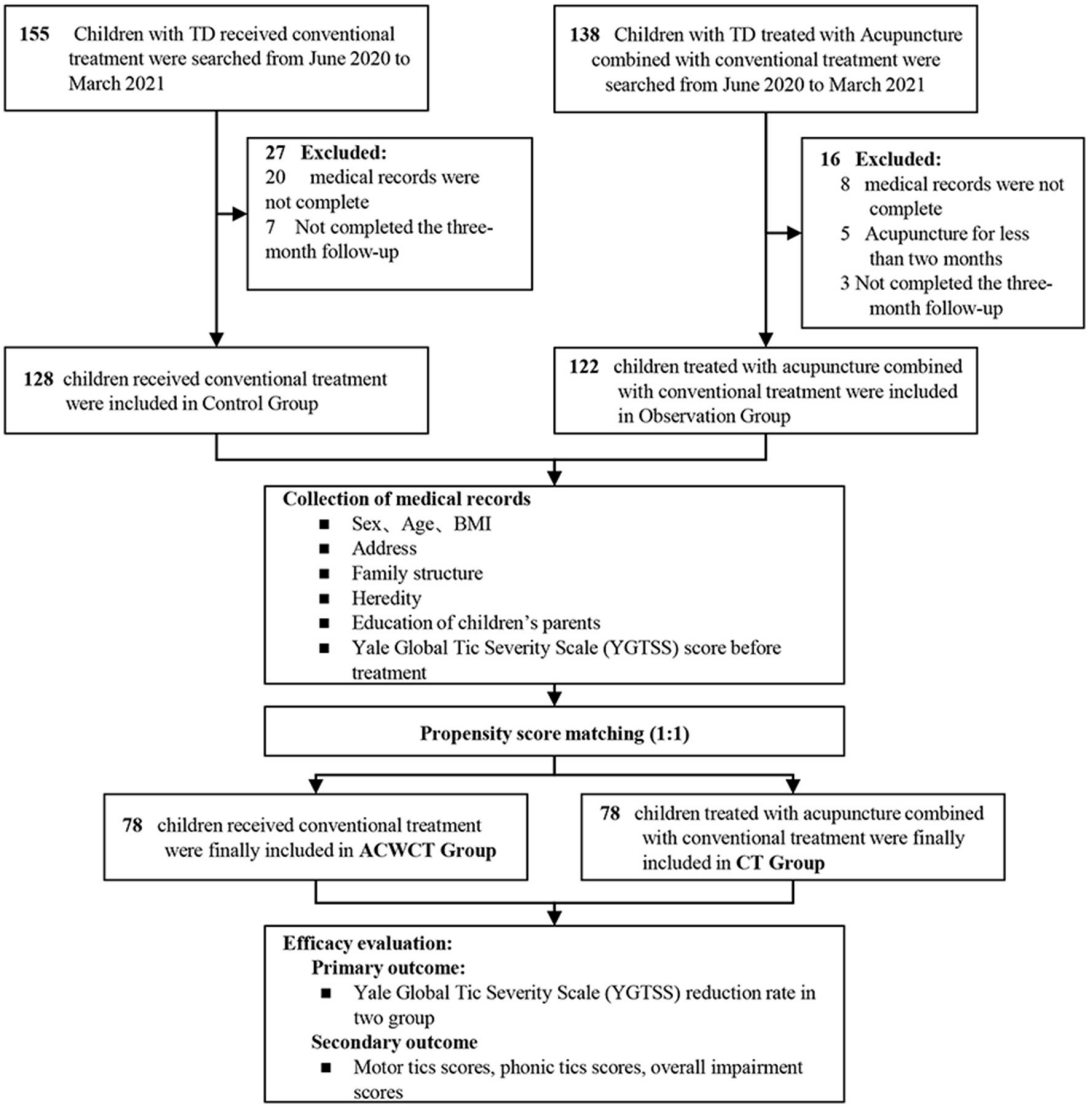
Study flow diagram. Observation group, acupuncture combined with conventional treatment. Control group, conventional treatment; BMI, body mass index.

This study was based in part on data collected from Traditional Chinese Medicine (TCM) clinics in a public pediatric hospital in Shanghai, between June 2020 and March 2021. Original data included information on demographic characteristics, medical care facilities, outpatient visits, visit dates, diagnostic codes, and prescriptions. We included 250 patients diagnosed with TD by using the Diagnostic and Statistical Manual of Mental Disorders, version 5 (DSM-5): 122 patients received acupuncture therapy combined conventional treatment (observation group) and 128 patients received conventional treatment only (control group). The patients were divided into the two groups by the actual treatments they had received. The choose of the treatments in the real clinical settings was a shared decision by the attending doctors and patients (their parents). After 1:1 propensity score matching, 78 patients for each group were included in the final analysis (Figure 1). Exclusion criteria were as follows: (1) TD accompanied by mental, behavioral, and developmental comorbidities, such as attention-deficit hyperactivity disorder (ADHD) or obsessive-compulsive disorder (OCD), depression and anxiety; (2) missing information for age and sex. Inclusion criteria were as follows: (1) patients who met the diagnostic criteria for TD in the DSM-5; (2) patients aged 4–16 years; (3) a (YGTSS) total score ≥14; and (4) patients treated with acupuncture therapy for at least 3 months. The progress of each patient's treatment was followed up by a professional doctor over the phone or in an outpatient clinic. The primary follow-up tool was the YGTSS, which was used to assess the severity of the patient's condition. This study was approved by the Ethics Committee of the Shanghai Children's Medical Center, Shanghai Jiao Tong University School of Medicine (SCMC IRB-K2019080-2). This study was conducted in accordance with the Declaration of Helsinki and its later amendments.

### Interventions

All patients were treated for more than 12 weeks, according to established medical protocols (14). The control group received clinical guidance based on standard conventional medical diagnosis and treatment for pediatric patients. Conventional treatment included psychological behavior therapy, medicine, and Massage alone or in combination. Some patients were given traditional Chinese medicine granules, adjusted based on the individual features of each patient (15). The observation group received acupuncture therapy with sterile single-use acupuncture needles (size 0.18 ×13 mm, manufactured by Suzhou Medical Products Co., Suzhou, China, Ltd.). The type of acupuncture was categorized into manual acupuncture, which can be identified by the treatment codes Ex-HN01 (Sishencong), GV24 (Shenting), ST8 (Touwei), and GB8 (Shuaigu). If the patient had facial symptoms, this treatment was supplemented with LI4 (Hegu). If the patient had symptoms of blinking and frowning, EX-HN3 (Yintang) and EX-HN4 (Yuyao) were added. If the patient presented with a strange sound in the throat, CV22 (Tiantu) and EX-HN4 (Yuyao) were sometimes implemented. Acupuncture points were named according to *Standard Acupuncture Nomenclature*.

The acupuncture procedures were conducted when the participants sat in chairs. After routine skin disinfection, nine sterile single-use acupuncture needles were inserted vertically for 0.5–2 mm until the patients experienced De qi (a sensation including sourness, numbness, tingling, aching, and a propagated feeling along the meridians). The needles were left in the acupoints for 20 min without any further manipulation. The ACWCT group received acupuncture three times a week. Acupuncture treatment was treated by an experienced acupuncture therapist.

### Outcome Measurement

The tic symptoms of each patient were evaluated on the YGTSS by an appointed professional that blinded to the interventions at baseline, and weeks 4 and 12 (16). The primary efficacy outcome measure was the reduction rate for the YGTSS total scores. These scores were compared from baseline (week 0) to post-treatment (weeks 4 and 12). The YGTSS rates motor and vocal tics separately on a 0–5 scale (where 0 = none and 5 = most severe), and within five domains (number, frequency, intensity, complexity, and interference); this results in a maximum possible score of 50 (17). Meanwhile, according to the YGTSS established by the American Academy of child psychiatry, patients' curative effect was evaluated on the reduction rate's judgment index (18). Reduction rate = [(pre-treatment scale score- post-treatment scale score)/pre-treatment scale score] ×100%. Basic recovery: score reduction rate of ≥ 95%. Remarkable effect: score reduction rate 70–95%, significantly improved symptoms. Effective: reduction rate 30%-70%, relieving symptoms. Invalid: score reduction rate <30%, the symptoms were not significantly improved or aggravated. The secondary outcome measure was the YGTSS entry score.

### Statistical Analysis

All statistical analyses were performed using Statistical Package for Social Sciences software, version 26.0 (SPSS, Inc., Chicago, IL, US), with a significance level of *p* = 0.05 and two-sided tests. Missing data were addressed by using multiple imputation. Descriptive statistics were provided to compare the characteristics of the two groups before and after matching. The 1:1 propensity score method was used to match an equal number of patients based on characteristics including sex, age, household registration, parental education level, family structure, body mass index, heredity, and YGTSS score; matching was conducted according to each patient's propensity score through nearest neighbor matching with a caliper value of 0.01. The method was used to reduce selection bias and influence of covariates on the comparisons. *T*-tests were used to analyze differences in characteristics between acupuncture users and non-acupuncture users. Multivariate logistic regression models were used to evaluate whether acupuncture therapy affected YGTSS reduction rates at weeks 4 and 12. Linear regression analyses were used to analyze the YGTSS total scores and score differences before and after treatment.

## Results

### Patients' Baseline Characteristics

Table 1 presents the patients' baseline characteristics before and after matching. Before matching, the two groups were significant different in residence location, highest education level of both parents, and YGTSS total score. After 1:1 propensity score matching, the patients' baseline characteristics of the two groups were similar. The proportion of boys was higher than that of girls in both groups, with an approximate male to female ratio of 3:1. Most patients had residence location of Shanghai, with parents having highest educational level of bachelor's degree and above, and from a complete family (lived with both biological parents).

**Table 1:** Patients' baseline characteristics before and after matching.

**Variable**	**Before PSM**		**After PSM**	
	**AT (*n* = 122)**	**NAT (*n* = 128)**	**AT (*n* = 78)**	**NAT (*n* = 78)**
Age (year)	8.59 ± 2.07	8.45 ± 2.01	8.27 ± 2.01	8.40 ± 2.14
Male (%)	94 (77.05)	102 (79.69)	59 (75.64)	59 (75.64)
BMI	17.88 ± 2.50	18.13 ± 2.91	17.83 ± 2.28	17.98 ± 3.07
From Shanghai	82 (67.21)	65 (50.78)^**^	49 (62.82)	51 (65.38)
Heredity	7 (5.74)	8 (6.25)	6 (7.69)	6 (7.69)
Father's HEL≥ Bachelor's degree	106 (86.89)	89 (69.53^**^	67 (85.90)	64 (82.05)
Mother's HEL≥ Bachelor's degree	100 (81.97)	89 (69.53)^*^	63 (80.77)	61 (78.21)
Family structure complete	112 (91.80)	124 (96.88)	76 (97.44)	74 (94.87)
YGTSS	25.25 ± 7.51	22.57 ± 7.19^*^	23.03 ± 6.45	23.10 ± 7.90

*Results were presented with mean ± SD or percentage. PSM, propensity score matching; AT, acupuncture treatment; NAT, non- acupuncture treatment; BMI, body mass index; HEL, highest educational level; YGTSS, the Yale Global Tic Severity Scale.*

* *P < 0.05;*

** *P < 0.01*.

### Improvements in YGTSS Scores

**Figure 2:**
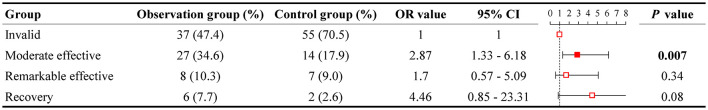
Comparison of YGTSS reduction rate after 4 weeks treatment after PSM (*n* = 78 for each group). Observation group, acupuncture combined with conventional treatment; Control group, conventional treatment. YGTSS, Yale Global Tic Severity Scale; OR, odds ratios; CI, confidence interval.

**Figure 3:**
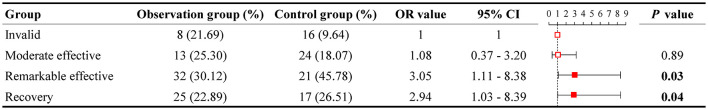
Comparison of YGTSS reduction rate after 12 weeks treatment after PSM (*n* = 78 for each group). Observation group, acupuncture combined with conventional treatment; Control group, conventional treatment. YGTSS, Yale Global Tic Severity Scale; OR, odds ratios; CI, confidence interval.

Figure 2 shows the comparative reduction rates between the two groups after 4 weeks of treatment. The moderate effective classification of the two groups was only statistically significantly different after 4 weeks of treatment (OR = 2.87, 95% CI = 1.33, 6.18, *P* = 0.007). Figure 3 presents a comparison of the efficacy classifications after 12 weeks of treatment. In contrast to the effect grade after 4 weeks of treatment, there were statistically significant differences in the remarkable effective rate and recovery rate at the end of the treatment period (OR = 3.05, 95% CI = 1.11, 8.38, *P* = 0.03; OR = 2.94, 95% CI = 1.03, 8.39, *P* = 0.04).

**Figure 4:**
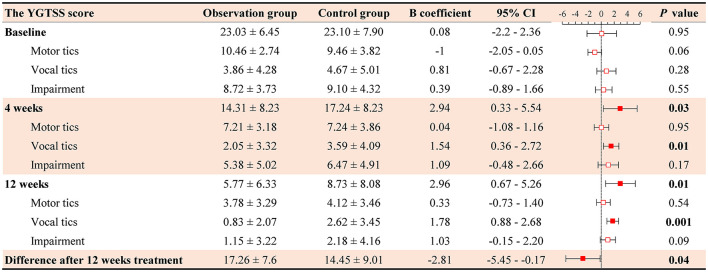
Comparison of the total YGTSS scores after 12 weeks treatment after PSM (*n* = 78 for each group). Observation group, acupuncture combined with conventional treatment; Control group, conventional treatment. YGTSS, Yale Global Tic Severity Scale; CI, confidence interval.

As presented in Figure 4, there was a statistically significant difference in YGTSS scores between the two groups at 4 and 12 weeks of post-treatment (β = 2.94, 95% CI = −0.33, 5.54, *P* = 0.03; β = 2.96, 95% CI = 0.67, 5.26, *P* = 0.01, respectively). Then, we analyzed the differences in total YGTSS scores between measurements at baseline and 12 weeks of treatment. Linear regression analyses demonstrated that clinical symptoms in the observation group were milder than those observed in the control group (β = −2.81, 95% CI = −5.45, −0.17, *P* = 0.04).

We further compared motor tics, vocal tics, and overall impairment rating scores between the two groups. The observation group had statistically significant differences in vocal tic scores against the control group (4 weeks: β = 1.54, 95% CI = 0.36, 2.72, *P* = 0.01; 12 weeks: β = 0.29, 95% CI = 0.88, 2.68, *P* = 0.001).

### Adverse Events

The treatment was done by a doctor specialist in acupuncture for Traditional Chinese Medicine. Regarding to minor reactions to treatment, some patients reported a momentary tingling pain, and the pain disappeared immediately after the acupuncture treatment. Also, few cases reported mild local bleeding, which was stopped within 1 min when pressed by medical cotton. Behavioral techniques such as demonstration, and distraction were applied to reduce the patient's fear of the procedure, and increase the friendliness and compliance.

## Discussion

The current study presented a preliminary examination of the efficacy of acupuncture treatment for children with TD in real clinical settings, using a propensity score-matched analysis. First, we found that the incidence of moderate efficacy was significant higher in the observation group than that in control group after 4 weeks of treatment. Second, patients treated with acupuncture combined with conventional therapy showed better recovery rates than those treated with conventional therapy alone after 12 weeks of treatment. Third, we observed that acupuncture treatment had additional beneficial effect in alleviating TD symptoms in children when combined with conventional treatment, especially for vocal tics. Considering the disadvantages and challenges of traditional treatments for tics in children as aforementioned (19, 20), our findings have significant implications in endorsing acupuncture as a complementary or alternative therapy for TD in children (21). In particular, as acupuncture has friendly accessibility and low cost in China, the current study is of great value for inspiring more efforts to search for its scientific base and underlying mechanisms, and scale-up its implementation.

Acupuncture is considered as an effective treatment method within TCM, but emerging studies indicate that acupuncture even has better short-term effect than pharmaceutical treatment for TS, and can improve the effect of pharmaceutical treatment on TS as an adjuvant therapy (22). Furthermore, acupuncture therapy may have fewer adverse effects and lower recurrence rates (23). Adding to existing literature on the efficacy of acupuncture for TD in children, the current study suggests that acupuncture is more efficacious for improving vocal motor tics, but has relatively limited efficacy for motor tics and overall impairments. At present, the mechanism for acupuncture in the treatment of TD in children is unclear. The vocal tics are mainly involving the muscles of mouth, throat, and nose. In TCM, vocal tic disorders can be attributed to the dysfunction of *zangfu* organs. The history of recurrent invasions of food irritation and infections results in excessive heat in the lung and stomach, which in turn causes the onset of vocal tics. Liver *qi* later transforms into fire, disturbing the mind, leading to the repetition of vocal tics (24). Therefore, it is possible that acupoint stimulation may improve the imbalance in heat disturbances in the body that causing vocal tics.

Current literature suggest a multifactorial etiology in TD, including immunological vulnerability, dysregulation of neurotransmission, and abnormal receptors (25, 26). Generally, acupuncture is often implemented to treat musculoskeletal problems of the four limbs, and attenuate inflammation through the vagus nerve that is mediated by dopamine (27, 28). Evidence suggest that acupuncture inhibits the production of IL-1 and other inflammatory cytokines *via* vagus nerve stimulation (29). Additionally, previous neuroimaging studies indicate that acupuncture may have neuromodulation effect (11, 12). Brain response to acupuncture stimuli encompasses a broad network of regions consistent with not just somatosensory, but also affective and cognitive processing, and executive functions (30). At present, whether the identified efficacy of acupuncture on TD in children works through these paths remains unclear and requires more investigations.

### Limitations and Strengths

Our study had several limitations. First, this is a retrospective study conducted in real clinical settings without pre-registered protocol, and thus the findings should be considered as preliminary, and require replication by randomized controlled trials in the future. Second, the lack of a same evaluator and the fact that the post-treatment evaluation was sometimes done over the phone could result in assessment bias. Third, placebo effects could lie in the control group and observation group, and would be hardly to exclude due to the study design. Beyond subjective measurements, future studies would better include biochemical data to support the observed findings and clarify the mechanism. Lastly, further efforts should examine whether the findings can be generalized to other health care systems such as Europe where acupuncture is not widely accessible and refundable. The study's main strengths are application of propensity score matching to balance baseline characteristics of the comparing groups, relatively large number of subjects, and long treatment duration. The findings of this retrospective study shed light on the efficacy of acupuncture for TD in children in real clinical settings, and should inspire future randomized clinical trials and studies exploring the underlying mechanisms.

## Conclusions

Our retrospective and propensity score-matched study provided preliminary evidence supporting the therapeutic effect of acupuncture for TD in children. The findings indicate that acupuncture could be an adjuvant treatment efficacious for TD in children, especially for vocal tics. Randomized controlled trials are needed to further investigate the efficacy and mechanisms of acupuncture in the treatment of TD in children.

## Data Availability Statement

The original contributions presented in the study are included in the article/Supplementary Material, further inquiries can be directed to the corresponding author/s.

## Ethics Statement

The studies involving human participants were reviewed and approved by Shanghai Children's Medical Center, which is affiliated with the Shanghai Jiao Tong University School of Medicine (SCMC IRB-K2019080-2). Written informed consent to participate in this study was provided by the participants' legal guardian/next of kin.

## Author Contributions

K-XS contributed to the overall conception and design of the study protocol. G-HW conceptualized and seriously edited the manuscript. H-ZY contributed to the specific study design and data analysis and wrote the manuscript's first draft. Y-FZ participated in the interpretation of clinical data. All authors have read, edited, approved the final manuscript, and agreed to be held accountable for aspects of the manuscript in ensuring that questions related to the accuracy or integrity of any part of the manuscript are appropriately investigated and resolved.

## Funding

This study was supported by the fund of Shanghai Administration of Traditional Chinese Medicine (ZHYY-ZXYJHZX-201918) [H-ZY (2021-2023)-0206-08], Shanghai Municipal Health Commission (2020LP022), and Shanghai Children's Medical Center (LY-SCMC2020-03).

## Conflict of Interest

The authors declare that the research was conducted in the absence of any commercial or financial relationships that could be construed as a potential conflict of interest.

## Publisher's Note

All claims expressed in this article are solely those of the authors and do not necessarily represent those of their affiliated organizations, or those of the publisher, the editors and the reviewers. Any product that may be evaluated in this article, or claim that may be made by its manufacturer, is not guaranteed or endorsed by the publisher.

## Abbreviations

TD: Tic disorders
TS: Tourette syndrome
CBIT: comprehensive behavioral intervention for tics
TCM: traditional Chinese medicine
ADHD: attention-deficit hyperactivity disorder
OCD: obsessive-compulsive disorder
YGTSS: Yale Global Tic Severity Scale
CT group: conventional treatment group
ACWCT group: acupuncture treatment combined with conventional treatment group
PSM: propensity score-matched.

## References

[1] Yang C Zhang L Zhu P Zhu C Guo Q. The prevalence of tic disorders for children in China: A systematic review and meta-analysis. Medicine. (2016) 95:e4354. 10.1097/MD.0000000000004354 27472724 PMC5265861

[2] Pringsheim T Nosratmirshekarlou E Doja A Martino D. Physical activity, sleep and neuropsychiatric symptom severity in children with tourette syndrome. Eur Child Adolesc Psychiatry. (2021) 30:711–9. 10.1007/s00787-020-01552-1 32372272

[3] Liu ZS Cui YH Sun D Lu Q Jiang YW Jiang L. Current status, diagnosis, and treatment recommendation for Tic disorders in China. Front Psychiatry. (2020) 11:774. 10.3389/fpsyt.2020.00774 32903695 PMC7438753

[4] Gargot T Arnaoutoglou NA Costa T Sidorova O Liu-Thwaites N Moorey S. Can we really teach cognitive behavioral therapy with a massive open online course? European psychiatry: the journal of the Association of European Psychiatrists. Eur Psychiatry. (2020) 63:e38. 10.1192/j.eurpsy.2020.29 32151289 PMC7358632

[5] Muller-Vahl KR Szejko N Verdellen C Roessner VJ Hoekstra P Hartmann A. European clinical guidelines for Tourette syndrome and other tic disorders: summary statement. Eur Child Adolesc Psychiatry. (2021). 10.1007/s00787-021-01832-4. [Epub ahead of print]. PMC8940881 34244849

[6] Dabrowski J King J Edwards K Yates R Hayman I Zimmerman-Brenner S. The long-term effects of group-based psychological interventions for children with tourette syndrome: a randomized controlled trial. Behav Ther. (2018) 49:331–43. 10.1016/j.beth.2017.10.005 29704964

[7] Liu JH Yan J Yi SX Chang XR Lin YP Hu JM. Effects of electroacupuncture on gastric myoelectric activity and substance P in the dorsal vagal complex of rats. Neurosci Lett. (2004) 356:99–102. 10.1016/j.neulet.2003.11.044 14746873

[8] Chen LY Yen HR Sun MF Lin CL Chiang JH Lee YC. Acupuncture treatment is associated with a decreased risk of developing stroke in patients with depression: A propensity-score matched cohort study. J Affect Disord. (2019) 250:298–306. 10.1016/j.jad.2019.03.020 30875672

[9] Yang CS Hao Z Zhang L-L Guo Q. Efficacy and safety of acupuncture in children: an overview of systematic reviews. Pediatric Res. (2015) 78:112–9. 10.1038/pr.2015.91 25950453

[10] Xiao L CY Yuanhao D Gao X Lin X Pan S. Evaluation of clinical randomized control trials of acupuncture for treatment of multiple Tics-coprolalia syndrome. Lishizhen Med Materia Medica Res. (2010) 21:1199–202. 10.3969/j.issn.1008-0805.2010.05.084

[11] Li M-K Li Y-J Zhang G-F Chen JQ Zhang JP Qi J. Acupuncture for ischemic stroke: cerebellar activation may be a central mechanism following Deqi. Neural Regene Res. (2015) 10:1997–2003. 10.4103/1673-5374.172318 26889189 PMC4730825

[12] Wong YK Wu JM Zhou G Zhu F Zhang Q Yang XJ. Antidepressant monotherapy and combination therapy with acupuncture in depressed patients: a resting-state functional near-infrared spectroscopy (fNIRS) study. Neurotherapeutics. (2021) 109:djw323. 10.1007/s13311-021-01098-3 34431029 PMC8804104

[13] Yao XI Wang X Speicher PJ Hwang ES Cheng P Harpole DH. Reporting and guidelines in propensity score analysis: a systematic review of cancer and cancer surgical studies. J Natl Cancer Inst. (2017) 109:323. 10.1093/jnci/djw323 28376195 PMC6059208

[14] Lu Q Sun D Liu ZS. Interpretation of expert consensus for diagnosis and treatment of tic disorders in China. Chin J Apply Clin Pediatric. (2021) 36:9. 10.3760/cma.j.cn101070-20201229-01967

[15] Rong P Ma R Han XM Wu HJ. Guideline for TCM pediatrics clinical diagnosis and treatment·tic disorder(amendment). J Pediatrics Traditional Chinese Med. (2019) 15:6. 10.16840/j.issn1673-4297.2019.06.01

[16] Storch EA De Nadai AS Lewin AB McGuire JF Jones AM Mutch PJ. Defining treatment response in pediatric tic disorders: a signal detection analysis of the Yale Global Tic Severity Scale. J Child Adolesc Psychopharmacol. (2011) 21:621–7. 10.1089/cap.2010.0149 22070181 PMC3279714

[17] Leckman JF Riddle MA Hardin MT Ort SI Swartz KL Stevenson J. The yale global Tic severity scale: initial testing of a clinician-rated scale of tic severity. J Am Acad Child Adolesc Psychiatry. (1989) 28:566–73. 10.1097/00004583-198907000-00015 2768151

[18] Martino D Pringsheim TM. Tourette syndrome and other chronic tic disorders: an update on clinical management. Expert Rev Neurotherapeutics. (2018) 18:125–37. 10.1080/14737175.2018.1413938 29219631

[19] Parraga HC Harris KM Parraga KL Balen GM Cruz C. An overview of the treatment of Tourette's disorder and tics. J Child Adolesc Psychopharmacol. (2010) 20:249–62. 10.1089/cap.2010.0027 20807063

[20] Waldon K Hill J Termine C Balottin U Cavanna AE. Trials of pharmacological interventions for Tourette syndrome: a systematic review. Behav Neurol. (2013) 26:265–73. 10.1155/2013/626410 22713420 PMC5215438

[21] Verdellen C van de Griendt J Hartmann A Murphy T. European clinical guidelines for Tourette syndrome and other tic disorders. Part III: behavioural and psychosocial interventions. Eur Child Adolesc Psychiatry. (2011) 20:197–207. 10.1007/s00787-011-0167-3 21445725

[22] Yu J Ye Y Liu J Wang W Peng WN Liu ZS. Acupuncture for tourette syndrome: a systematic review. Evid Based Complement Alternat Med. (2016) 2016:1834646. 10.1155/2016/1834646 27725839 PMC5048029

[23] Lu C Wu L-Q Hao H Kimberly Leow XT Xu FW Li PP. Clinical efficacy and safety of acupuncture treatment of TIC disorder in children: A systematic review and meta-analysis of 22 randomized controlled trials. Complement Therap Med. (2021) 59:102734. 10.1016/j.ctim.2021.102734 33989798

[24] Duan LN Su SY Xu YF Li M. Thirty cases of childhood vocal tic disorders treated with acupoint catgut embedding combined with auricular plaster therapy (sic). World J Acupuncture-Moxibustion. (2021) 31:55–8. 10.1016/j.wjam.2020.11.009

[25] Landau YE Steinberg T Richmand B Leckman JF Apter A. Involvement of immunologic and biochemical mechanisms in the pathogenesis of Tourette's syndrome. J Neural Transmission. (2012) 119:621–6. 10.1007/s00702-011-0739-x 22139323 PMC3936959

[26] Martino D Dale RC Gilbert DL Giovannoni G Leckman JF. Immunopathogenic mechanisms in tourette syndrome: A critical review. Movement Disord. (2009) 24:1267–79. 10.1002/mds.22504 19353683 PMC3972005

[27] Torres-Rosas R Yehia G Peña G Mishra P Thompson-Bonilla MDR Moreno-Eutimio MA. Dopamine mediates vagal modulation of the immune system by electroacupuncture. Nat Med. (2014) 20:291–5. 10.1038/nm.3479 24562381 PMC3949155

[28] Cox J Varatharajan S Côté P Collaboration O. Effectiveness of acupuncture therapies to manage musculoskeletal disorders of the extremities: a systematic review. J Orthopaedic Sports Phys Ther. (2016) 46:409–29. 10.2519/jospt.2016.6270 27117725

[29] Kavoussi B Ross BE. The neuroimmune basis of anti-inflammatory acupuncture. Integrat Cancer Therap. (2007) 6:251–7. 10.1177/1534735407305892 17761638

[30] Huang W Pach D Napadow V Park K Long XY Neumann J. Characterizing acupuncture stimuli using brain imaging with FMRI–a systematic review and meta-analysis of the literature. PLoS ONE. (2012) 7:e32960. 10.1371/journal.pone.0032960 22496739 PMC3322129

