# Supplementary material for: The Efficacy of Acupuncture on Tic Disorders in Children: A Retrospective and Propensity Score-Matched Study
Source: Front Pediatr. 2021 Nov 4;9:745212. doi: 10.3389/fped.2021.745212 (PMC8600324; doi:10.3389/fped.2021.745212)
Supplement: Supplementary file 2 [file Table_2.DOCX]

**SUPPLEMENTARY MATERIALS**

| **Supplementary Table.** Full guidelines for reporting propensity score analysis, modified From the STROBE (Strengthening the Reporting of observational studies in Epidemiology) Statement* | | | |
| --- | --- | --- | --- |
| **Section/topic** | **Item No** | | **Recommendation** |
| **Title and abstract** | **□** | 1 | Indicate the use of propensity analysis with a commonly used term in the title or the abstract |
|  | **□** | 2 | Provide in the abstract an informative and balanced summary of what was done and what was found |
| **Introduction** |  |  |  |
| Background/rationale | **□** | 3 | Explain the scientific background and rationale for the investigation being reported |
| Objectives | **□** | 4 | State specific objectives, including any prespecified hypotheses |
| **Methods** |  |  |  |
| Setting | **□** | 5 | Describe the setting, locations, and relevant dates, including periods of recruitment, treatment, follow-up, and data collection |
| Patient selection | **□** | 6 | Give the eligibility criteria, and the sources and methods of subject ascertainment and selection |
| Variables | **□** | 7 | Clearly define all outcomes, treatments, predictors. Give diagnostic criteria, if applicable |
| Data sources/ measurement | **□** | 8 | For each variable of interest, give sources of data and details of methods of assessment (measurement) |
| Bias | **□** | 9 | Describe how propensity score analysis was used to address bias |
|  | **□** | 10 | Describe any other methods to address potential sources of bias, e.g. sensitivity analysis |
| Sample size | **□** | 11 | Explain how the study size was arrived at |
| Statistical analyses | **□** | 12 | Describe all the analytic methods, including the propensity score methods, e.g. matching, weighting, stratification, or covariate adjustment using propensity score |
|  | **□** | 13 | Indicate the model used to estimate propensity score, e.g. logistic model, boosting (meta-classifiers), decision trees |
|  | **□** | 14 | State the variables included in the propensity score model |
|  | **□** | 15 | Explain the variable selection procedure for propensity score model |
|  |  | 16 | For propensity score matching: |
|  | **□** | 16.1 | Explicitly state the matching algorithm and distance metric |
|  | **□** | 16.2 | Indicate matching ratio (1:m matching) |
|  | **□** | 16.3 | Indicate whether sampling with or without replacement was used |
|  | **□** | 16.4 | Describe the statistical methods for the analysis of matched data |
|  | **□** | 16.5 | Describe methods for assessing the comparability of baseline characteristics in the matched groups |
|  | **□** | 17 | For propensity score weighting, describe methods for assessing the comparability of baseline characteristics in the weighted groups |
|  |  | 18 | For propensity score stratification: |
|  | **□** | 18.1 | Give the number of strata |
|  | **□** | 18.2 | Describe methods for assessing the comparability of baseline characteristics in each stratum |
|  | **□** | 19 | Explain how assumption of propensity score analysis was examined |
|  | **□** | 20 | Explain how missing data were addressed, including missing data in propensity score estimation |
|  | **□** | 21 | If applicable, describe any methods used to examine subgroups and interactions |
|  | **□** | 22 | Describe any sensitivity analyses |
|  | **□** | 23 | Indicate the software used for analysis |
|  | **□** | 24 | If applicable, report the package used to create matched sample, e.g. GMATCH macro in SAS, MatchIt package®, Optmatch package ® |
| **Results** |  |  |  |
| Participants |  | 25 | Report numbers of participants at each stage of study: |
|  | **□** | 25.1 | sample size of patients potentially eligible |
|  | **□** | 25.2 | sample size of patients confirmed eligible and included |
|  | **□** | 25.3 | sample size of patients analyzed |
|  | **□** | 25.4 | for propensity score matching, sample size for each treatment group before and after matching |
|  | **□** | 26 | Explain reasons for exclusion at each stage |
|  | **□** | 27 | Consider use of a flow diagram |
| Patient characteristics | **□** | 28 | Describe the distribution of baseline characteristics for each group before propensity score analysis |
|  |  | 29 | For propensity score matching, weighting, or stratification: |
|  | **□** | 29.1 | Desc Describe the distribution of baseline characteristics in the matched/weighted groups or in each stratum |
|  | **□** | 29.2 | Describe the results of the comparability of baseline characteristics, whether there are still systematic differences between treatment groups |
|  | **□** | 30 | Indicate number of patients with missing data for each variable of interest, especially the variables used  in propensity score model |
| Outcome data | **□** | 31 | Report outcomes of each treatment group |
| Main results | **□** | 32 | Give propensity score analysis estimates and their precision, e.g. 95% confidence interval |
|  | **□** | 33 | If applicable, give unadjusted estimates and/or adjusted estimates and their precision, e.g. 95% confidence interval. Make clear which additional factors were adjusted for |
| Other analyses | **□** | 34 | Report other analyses done, e.g. analyses of subgroups and interactions, and sensitivity analyses |
| **Discussion** |  |  |  |
| Key results | **□** | 35 | Summarize key results with reference to study objectives |
| Limitations | **□** | 36 | Discuss limitations of the study, taking into account sources of potential bias or imprecision |
|  | **□** | 37 | Discuss both direction and magnitude of any potential bias |
| Interpretation | **□** | 38 | Discuss whether imbalance of baseline characteristics still exists, and give a cautious interpretation |
|  | **□** | 39 | Give a cautious overall interpretation of results considering objectives, limitations, multiplicity of analyses, results from similar studies, and other relevant evidence |
| Generalizability | **□** | 40 | For propensity score matching, discuss the possibility and potential influence of incomplete matching, especially the studies in which the matched sample size is less than 50% |
| **Other information** |  |  |  |
| Funding | **□** | 41 | Give the source of funding and the role of the funders for the present study and, if applicable, for the original study on which the present article is based |

* von Elm E, Altman DG, Egger M, et al. The Strengthening the Reporting of Observational Studies in Epidemiology (STROBE) statement: guidelines for reporting observational studies. J Clin Epidemiol 2008;61(4):344-9.

This guideline can be downloaded at:

<https://sites.duke.edu/xiaofeiwang/files/2016/12/Supplementary-Table-6.pdf>
